# Cathepsin S Cleaves BAX as a Novel and Therapeutically Important Regulatory Mechanism for Apoptosis

**DOI:** 10.3390/pharmaceutics13030339

**Published:** 2021-03-05

**Authors:** Surinder M. Soond, Lyudmila V. Savvateeva, Vladimir A. Makarov, Neonila V. Gorokhovets, Paul A. Townsend, Andrey A. Zamyatnin

**Affiliations:** 1 Institute of Molecular Medicine, Sechenov First Moscow State Medical University, Trubetskaya Str. 8-2, 119991 Moscow, Russia; ludmilaslv@yandex.ru (L.V.S.); known.sir@yandex.ru (V.A.M.); gorokhovets_n_v@staff.sechenov.ru (N.V.G.); 2 Division of Cancer Sciences and Manchester Cancer Research Centre, Faculty of Biology, Medicine and Health, University of Manchester, Manchester M13 9PL, UK; paul.townsend@manchester.ac.uk; 3 Faculty of Health and Medical Sciences, University of Surrey, Guildford, Surrey GU2 7X, UK; 4 Belozersky Institute of Physico-Chemical Biology, Lomonosov Moscow State University, 119992 Moscow, Russia; 5 Department of Biotechnology, Sirius University of Science and Technology, 1 Olympic Ave, 354340 Sochi, Russia

**Keywords:** cathepsins, BAX, MOMP, ubiquitination, apoptosis, chemotherapy, cancer

## Abstract

Certain lysosomal cathepsin proteins have come into focus as being good candidates for therapeutic targeting, based on them being over-expressed in a variety of cancers and based on their regulation of the apoptotic pathway. Here, we report novel findings that highlight the ability of cathepsin S expression to be up-regulated under Paclitaxel-stimulatory conditions in kidney cell lines and it being able to cleave the apoptotic p21 BAX protein in intact cells and in vitro. Consistent with this, we demonstrate that this effect can be abrogated in vitro and in mammalian cells under conditions that utilize dominant-inhibitory cathepsin S expression, cathepsin S expression-knockdown and through the activity of a novel peptide inhibitor, CS-PEP1. Moreover, we report a unique role for cathepsin S in that it can cleave a polyubiquitinated-BAX protein intermediate and is a step that may contribute to down-regulating post-translationally-modified levels of BAX protein. Finally, CS-PEP1 may possess promising activity as a potential anti-cancer therapeutic against chemotherapeutic-resistant Renal Clear Cell Carcinoma kidney cancer cells and for combined uses with therapeutics such as Paclitaxel.

## 1. Introduction

The lysosomal cathepsin proteases are emerging as being quite pleiotropic concerning the regulatory role they play in a number of key cellular processes that are central to disease progression [1]. From this family of proteins, the cysteine cathepsins have gained considerable attention due to their ability to be auto-activated, remain catalytically active at lysosomally-acidic [2] and cytoplasmically-neutral pHs and include cathepsins -B, -C, -F, -H, -K, -L, -O, -S, -V, -X, -W and -Z [1,3]. More recently, cathepsin proteases have gained in importance based on their contributions to cancer progression and angiogenesis, when expressed extracellularly or intracellularly, and have been evaluated for their candidacy as therapeutic targets [1,3,4,5,6,7,8]. One such member from this hopeful family of targets is cathepsin S (CS), particularly in the context of cardiovascular disease or gastric- and colon-cancers [9].

Mechanistically, the cathepsins gained importance following the seminal studies, which demonstrated the ability of cathepsins -B, -K, -L, -H, -D and possibly -S to cleave and activate the Bcl-2 family member BID or caspase-8, resulting in their respective activation of the intrinsic and extrinsic arms of the apoptosis pathway [10,11]. Briefly, the Bcl-2 family of proteins can be divided into 3 sub-families: pro-apoptotic (for example BAX and BAK), anti-apoptotic (which include Bcl-2 and Bcl-xl), and the BH3-only proteins (such as BID, BAD and BIM) [12,13]. Of importance is the p21 BAX protein, which is structurally composed of 9 α-helices [14], is predominantly monomeric [15] and is activated through two possible mechanisms upon stimulation of cells with cytotoxic agents [16]. Firstly, binding of BH3-only proteins to helices α1–α6 (the ‘rear pocket’ of p21 BAX) can displace the α-helix 9 *trans*-membrane (TM) domain from the hydrophobic groove (composed of helices α2–α5), thus allowing the insertion of the TM domain into the outer mitochondrial membrane leading to mitochondrial outer membrane permeabilization (MOMP) [17]. Secondly, displacement of the α1–α2 helices by activator BH3-proteins can expose the BH3 domain [18] and permit the oligomerization of BAX [18,19], which is a key step in activating MOMP and apoptosis through the intrinsic pathway [20].

The BH3 domain of p21 BAX can also bind the hydrophobic groove of the inhibitory Bcl-2 protein (through helices α2–α5 [21]), which reduces the ability of p21 BAX to oligomerize to 80-, 96-, 160- or 260-kD complexes [22], thus limiting BAX-mediated MOMP activation [23]. Therapeutically, targeting this *pro*-apoptotic BH3 domain protein-*anti*-apoptotic Bcl-2 protein hydrophobic groove interaction using ‘BH3 mimetics’ has yielded a number of very promising *anti*-cancer drugs such as Venetoclax [24,25] and WEHI539 [26].

Based on the importance of cathepsin-mediated cleavage of BH3-domain containing pro-apoptotic proteins, albeit an area of controversy [27,28], we questioned the regulatory dynamics that exist between the CS and p21 BAX proteins in the context of kidney cancer. Here, we report that cathepsin S (*CTSS*) and *BAX* gene expression through Paclitaxel stimulation or cellular stress are inducible in kidney cell lines. We also report that p21 BAX is a novel substrate for CS, which may be a step that is important in regulating apoptosis as it has the effect of depleting cells of the p21 BAX protein. Moreover, we show that this cleavage event can be inhibited in the presence of a novel Bcl-xl protein-derived inhibitory peptide (named CS-PEP1) and which can induce apoptosis of HEK293 and 769P cancer cells through mechanisms that involve p21 BAX protein stabilization and Bcl-xl protein destabilization. Collectively, our novel findings offer good potential for Paclitaxel-resistant Renal Clear Cell Carcinoma (RCCC) cancer cells [29] to be sensitized to the apoptosis-inducing effects of CS-PEP1 alone or in combination with Paclitaxel therapy.

## 2. Materials and Methods

**Reagents, Antibodies and cells**

Paclitaxel (Pac, in DMSO), hydrogen peroxide (HP), z-VAD-FMK (in DMSO), MG132 (in DMSO) and 100 mM cycloheximide (in DMSO) were purchased from (Sigma-Aldrich, Moscow, Russia). Mouse *anti*-human Cathepsin S (E-3) was purchased from Santa Cruz Biotechnology (Santa Cruz, CA, USA), and *anti*-HA tag (SG77) or goat *anti*-Cathepsin S (PA5-47088) were purchased from Invitrogen (Carlsbad, CA, USA). *Anti*-Bcl-xl (Ab15274) and -BAX (Ab3154) were purchased from Abcam (Cambridge, UK). An *anti*-active caspase-3 specific antibody was purchased from Abcam (Ab13847) to detect the cleaved 17 kD form of caspase-3 by Western blotting. Protein AG agarose was purchased from Pierce (Waltham, MA, USA). HEK293 (RRID:CVCL_0045) and 769-P (RRID:CVCL_1050) kidney cells lines were sourced originally from ATCC and respectively maintained in DMEM (Invitrogen, Carlsbad, CA, USA) and RPMI 1640 (Invitrogen, Carlsbad, CA, USA) medium containing 10% FBS and antibiotics, as recommended by the supplier (Invitrogen, CA, USA). All human cell lines were authenticated using Short Tandem Repeat (STR) profiling within the last three years. All experiments were performed with mycoplasma-free cells. The Bcl-xl-derived peptide CS-PEP1 (of sequence FFSFGGAL) was synthesized by Peptech (Saint Petersburg, Russia) and dissolved in sterile DMSO as a stock solution of 10 mM.

**Expression Plasmids and Primers**

Cathepsin S-FLAG (pCS-FLAG) and cathepsin S C25A-FLAG (pCS-C25A-FLAG) mammalian expression plasmids were gifts from Dr Yun-Sin Lee [30]. BID, BAX, Bcl-xl coding sequences were PCR cloned into pCDNA3.1+/HA for carboxyl-terminal HA-epitope tagging. Bcl-xl-HA was difficult to detect using Western blotting, possibly due to c-terminal processing of the protein. Plasmid pBAX was made by cleaving pBAX-HA with *Xho* I, T_4_ DNA polymerase fill-in reaction, and auto-ligation (to remove in-frame HA-tag expression). The coding sequences for BAX and Bcl-xl were respectively sub-cloned into pET28A and pGEX5x-1 for bacterial expression. Bcl-2 protein and CS PCR primers:

BAX-GCTAGCTGAATTCATGGACGGGTCCGGGGAGCAG and CCGCTCGAGGCCCATCTTCTTCCAGATGG;

BID-GCTAGCTGAATTCATGGACTGTGAGGTCAACAACGGTTCC and CCGCTCGAGGTCCATCCCATTTCTGGCTAAGCTCC;

Bcl-xl-GCTAGCTGAATTCATGTCTCAGAGCAACCGGGAGCTGGTGG and CCGCTCGAGTTTCCGACTGAAGAGTGAGCCC.

The human cathepsin s coding sequence was PCR cloned into pET15b using: CATHS-TATACATATGCAGTTGCATAAAGATCCTACC and CATH-AS TTCTCGAGCTAGATTTCTGGGTAAGAGG.

**5′ *CTTS* gene reporter construction**

The -1048 bp and -564 bp fragments upstream of the cathepsin S (CS) gene sequence were PCR cloned using:-1048 bp (CTCGAGTGCACTCCAGCATGGGAGACAAA with GTCAATTGAACTGAAATAAGCTT); and -564 bp (CTCGAGTACAAGGAGCTGGGATTTGGGG with GTCAATTGAACTGAAATAAGCTT). DNA fragments were subcloned into *Xho* I/*Hind* III digested pTurbo-GFP PRL, giving pCS-1048 and pCS-564. Mammalian cells were transfected with 1 μg of plasmid for 24 h and stimulated with Pac (5 μg/mL) for a further 24 hr, lysed and equal volumes analyzed by Western blotting for GFP expression, using an *anti*-GFP antibody (Evrogen, Moscow, Russia).

**Semi-Quantitative RT-PCR**

Cells seeded on 100 mm tissue culture plates were stimulated as outlined and total RNA isolated using Trizol reagent (Invitrogen, CA, USA). 1 μg of total RNA was reverse transcribed MMLV Reverse Transcriptase kit (Evrogen, Moscow, Russia). 2.5 ng of cDNA template was used in the qPCRmix-SH system (Evrogen, Moscow, Russia) for each reaction, at an annealing temperature of 55 °C and equal volumes (10 μL) analyzed on 1% agarose gels. Primers used to quantify CS, BAX and GAPDH transcripts were: CS-F (TCAACTGAAAAATATGGAA), CS-R (CCTTCTTCACCAAAGTTGTGGCC), BAX-F (ATGTTTTCTGACGGCAACTTC), BAX-R (AGTCCAATGTCCAGCCCAT), GAPDH-F (CTTCGCTCTCTGCTCCTGTTCG) and GAPDH-R(ACCAGGCGCCCAATACGACCAAAT), and gave 390, 133, and 140 bp products, respectively.

**Primers for CS knockdown plasmid construction**

The pCS-shRNA expression plasmid was made by annealing the two primers of the sequence;GATCGGGAGACATGACCAGTGAAGATCAAGAGTCTTCACTGGTCATGTCTCCC and GATCGGGAGACATGACCAGTGAAGACTCTTGATCTTCACTGGTCATGTCTCCC, followed by their ligation into *Bgl* II digested plasmid pTER (as outlined in [31]). Insert integrity was confirmed by DNA sequencing, and approximately 3 μg plasmid was used to transfect HEK293 or 769P cells for 24 h, before cells were lysed and equal volumes of cleared lysates analyzed by reducing SDS-PAGE/Western blotting.

**Bacterial expression and purification of proteins**

Bacterial strain BL-21 (DE3) Lys-S was used for expression of bacterial BAX-His_6_ and GST-Bcl-xl fusion proteins. GST-Bcl-xl protein was purified as outlined previously [32] and the BAX-His_6_ was bacterially expressed, batch purified using the method outlined previously [31] and the protein kept immobilized on NTA agarose (Pierce, Waltham MA, USA) for subsequent CS-mediated cleavage assays. Cathepsin S protein was expressed in BL-21 cells, sonicated and inclusion bodies solubilized in 4 mL 20 mM Tris-HCl, 6 M Guanidine hydrochloride (Gnd-HCl), 0.5 M NaCl and 10 mM imidazole (Im), pH 8.0 (Buffer A). Cleared lysates were applied to an IMAC column, washed in Buffer A and eluted using 6 M Gnd-HCl, 0.5 M NaCl, 0.3 M Im, pH 8.0 (Buffer B). DTT was added to a final concentration of 10 mM to eluted fractions, which were dialyzed against refolding buffer (1 M Tris-HCl, 1 M Arg-HCl, 1 mM reduced L-Glutathione, 2 mM oxidized L-Glutathione, 2 mM EDTA, pH 8.0). Cleared protein solution was concentrated using a 10 kD cut-off Amicon protein concentrating cell and protein concentration was determined.

**Transfection of mammalian cells**

HEK293 or 769P cells were seeded at 1.5 × 10^5^ cells per 35 mm well and transfected using the recommended amount of plasmid DNA with Turbofect reagent (Thermo Fisher Scientific, Carlsbad, CA, USA). Generally, pBAX-HA (or other Bcl-2 protein encoding plasmids) were co-transfected at 0.5 μg with 1.5 μg pCS-FLAG (or pCS-C25A-FLAG) or at a respective ratio of 1:3 per transfection of HEK293 or 769P cells. Plasmid pCDNA3.1+ was used in negative control transfections and to keep plasmid amounts consistent between differing transfection conditions. Transfected cells were lysed in equal volumes of 1% NP40LB (as described in [33]) and equal volumes of cleared whole cell lysates used in subsequent procedures.

**Cycloheximide Pulse Chase Studies**

Cells were transfected for 24 h with 0.25 μg BAX-HA and CS-FLAG-derived expression plasmids (0.75 μg) for 6 h and left either untreated or treated with 10 μM CS-PEP1 for a further 18 h. Cells were then treated with 20 μM cycloheximide (in medium containing 10% serum) for 1 h and the cells washed in PBS, lysed in 1% NP40LB buffer at the indicated time points and 10% *v*/*v* of cleared whole cell lysate analyzed by reducing SDS-PAGE and Western blotting.

**Immunoprecipitation of Proteins**

These were carried out overnight as described previously [31,33], with 0.25 μg of *anti*-HA antibody and 30 μL of protein AG agarose beads (Pierce).

**Cathepsin S-Ni-NTA Co-precipitations**

30 μL of Ni-NTA agarose (Pierce) were incubated with 5 μg of CS protein diluted in 1 mL 1% NP40LB (lacking EDTA) and incubated with gentle rotation overnight at 4 °C. The resulting CS-NTA agarose was washed six times with 1% NP40LB (-EDTA) using cycles of microcentrifugation and after the last wash, equal volumes of 100 mM sodium acetate pH 4.0 buffer (50 μL) added and the mix incubated at 37 °C for 5 min. 50 μL of 100 mM Na_2_PO_4_, pH 7.2 were added, followed by cleared lysate prepared from mammalian cells expressing BAX-HA and the resulting mixtures incubated at 4 °C overnight with gentle rotation. As a negative control, Ni-NTA alone was also incubated with equal volumes of lysate. Precipitates were washed using 1 mL 1% NP40LB (-EDTA) six times and then 20 μL of 2× Laemmli buffer/DTT added followed by heating to 95 °C for 5 min. Samples were analyzed by Western blotting using the *anti*-HA antibody to detect BAX-HA. 5% *v*/*v* of the cleared lysate expressing BAX-HA was also analyzed by Western blotting.

**Isolation of ubiquitinated BAX intermediates**

p21 BAX protein (pBAX-3.1+, 0.25 μg) was co-expressed with HA-tagged ubiquitin (0.5 μg) or cathepsin S-FLAG derivatives (0.75 μg) in mammalian cells and cleared soluble lysates prepared using 1% NP40LB containing protease inhibitors. Immune complexes were purified using *anti*-HA antibody overnight, washed and 50 μL of 2× Laemmli buffer added to the beads before samples were boiled for 5 min. 950 μL of 1% NP40LB were added to each sample, centrifuged and the soluble fraction removed and immunoprecipitated again, overnight using the *anti*-HA antibody (as outlined above) before being washed six times with 1% NP40LB and equal volumes analyzed by reducing SDS-PAGE and Western blotting, as described [31] (either before or after being subjected to cathepsin S-mediated cleavage, in vitro).

**In vitro cathepsin S cleavage assays**

Purified immune complexes (as described in [31]) were washed in 1 mL of 100 mM Na_2_PO4, pH 7.2 buffer and resuspended in 20 μL of the same buffer. An equal volume of 100 mM sodium acetate (pH 4.0) buffer was added to 0.5 μg of purified cathepsin S and the mixture incubated at 37 °C for 10 min to auto-activate the cathepsin S protein. An equal volume of 100 mM Na_2_PO_4_ pH 7.2 buffer was added to this enzyme mix giving a final concentration of cathepsin S of 0.125 μg/μL. For pH 7 cleavage assays, 4 μL of this mix (0.5 μg cathepsin S) were added to the immunoprecipitated complexes, 100 ng BAX-Ni-NTA agarose beads or 0.5 μg GST-BCl-xl agarose beads that had been previously washed twice in 1 mL of 100 mM Na_2_PO_4_, pH 7.2 and then resuspended in 20 μL of 100 mM Na_2_PO_4_, pH 7.2, 2 mM DTT. The reactions were incubated at 37 °C for 50 min and the reaction terminated upon the addition of 20 μL of 2× Laemmli Buffer (containing 100 mM DTT), followed by a 5 min incubation at 94 °C. For cleavage assays conducted at pH 5, immobilized substrate proteins (in 20 μL of 100 mM Na_2_PO_4_ buffer, pH 7.2) were resuspended in 20 μL of 100 mM sodium acetate (pH 4.0), prior to the addition of 0.5 μg (4 μL) of activated cathepsin S mix, followed by incubation at 37 °C for 50 min and termination with 2× Laemmli Buffer/DTT and heating to 95 °C for 5 min. Reaction mixtures were scaled up so that their pH could be determined with greater accuracy using a pH electrode (Millipore). Equal reaction volumes were analyzed by SDS PAGE or Western blotting.

**Laser Scanning Confocal Microscopy**

HEK293 cells were seeded on glass coverslips, transfected for 24 h and then stimulated with Pac (or HP) for an additional 24 h. Coverslips were fixed in 4% PFA/PBS pH 7.4, permeabilized in 0.1% Triton X-100/PFA/PBS, washed and treated with 100 mM Glycine PBS/pH 8.2 for 30 min and incubated in primary antibody (at the recommended dilution) for 1 h. Incubation in secondary antibody-fluorophore conjugate (Molecular probes, Eugene, OR, USA) was for 1 h and coverslips mounted in Mounting Mix Gold-with or without DAPI stain (Invitrogen). Images were recorded on a Zeiss LSM880 Airyscan microscope using Zeiss Zen Black software.

**Apoptosis Assays**

HEK293 or 769P cell lines were treated as outlined in the Click-IT TUNEL Alexa-Fluor 594 Imaging Assay kit (Molecular Probes) and coverslips mounted on microscope slides in the presence of DAPI staining. Between 200–1000 cells were visualized using 20x and 40x objectives on a Zeiss LSM 880 Airyscan laser scanning confocal microscopy and apoptotic cells (stained in red) scored. Results from one series of experiments are presented where consistency was seen between duplicated experiments. Experiments were repeated identically in the absence of seeding cells on coverslips and cells lysed in 200 μL of 1% NP40LB and 2× Laemmli buffer/DTT before samples were heated at 95 °C for 10 min for Western blot analysis.

**Statistical Analysis and Densitometry**

Experiments were performed independently with an *n* = 3. The results representative of one experiment are highlighted within the figures, where consistency was seen at least twice. Western blot images were analyzed using Adobe Photoshop CS4 to quantify and record the intensity of bands (using technical repetitions of *n* = 3, which were generally all normalized for β-actin protein levels as observed for unstimulated HEK293 or 769P cells (and negative control samples) using Excel (Microsoft, Moscow, Russia). For statistical analyses, the intensity of bands (or apoptotic cell numbers) were calculated from the mean between three readings and the Standard Error around the Mean (±SEM). These results were plotted using Excel (Microsoft, Moscow, Russia). The two-tails unpaired student *t-test* (Excel) was performed to test for significance of ±SEM values (Excel). Source data are available for all figures.

## 3. Results

### 3.1. The CTTS Gene Is Induced under Paclitaxel or Hydrogen Peroxide Stimulatory Conditions

**Figure 1:**
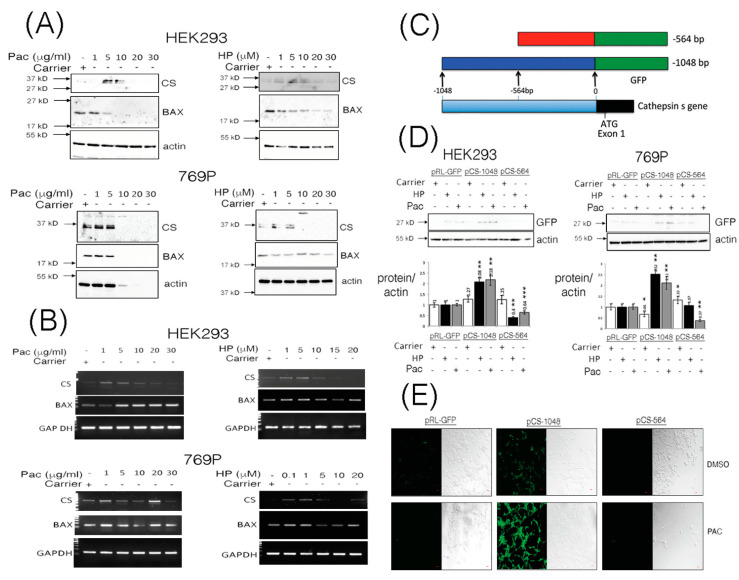
Cathepsin S (CS) protein expression levels are induced through the -1048 promoter fragment from the *CTTS* gene upon Paclitaxel or hydrogen peroxide stimulation. HEK293 and 769P cells were stimulated with increasing doses of Paclitaxel (Pac) and hydrogen peroxide (HP) and soluble lysates prepared after 24 h and equal volumes analyzed by Western blotting for cathepsin S (CS), BAX and actin expression (Panel **A**). Similarly, total RNA was isolated from cells stimulated for 24 h with increasing doses of Pac or HP and equal quantities of template cDNA analyzed for transcriptional expression of CS, BAX and Glyceraldehyde-3-phosphate dehydrogenase (GAP-DH, Panel **B**). Promoter fragments, derived from the transcriptional start site of the *CTTS* gene to -1048 and -564, were PCR cloned and fused to a promoter-less GFP encoding plasmid (Panel **C**) and evaluated for GFP expression in equal volumes of HEK293 and 769P cleared whole cell lysates (WCLs) under Pac (10 μg/mL and 5 μg/mL, respectively) or HP (5 μM) stimulatory conditions. GFP expression was also quantified, standardized and corrected against GFP expression from cells stimulated with carrier alone and is shown as a fold change over basal GFP expression (Panel **D**). HEK293 cells transfected with pCS-1048 or pCS-564 and stimulated with Pac (5 μg/mL) for 24 h were also fixed and visualized for GFP expression using laser scanning confocal microscopy (Panel **E**). The red bar indicates 1 micron. Quantified data are presented as the mean ± SEM and its significance (where *p* < 0.05) determined, using a two-way Student’s *t*-test (* *p* < 0.05, ** *p* < 0.01 and *** *p* < 0.001).

While the classical approach to defining a direct relationship between cathepsins and cancer have emerged through characterizing deregulated expression of cathepsins in tumor progression [1], relatively less is understood about the inducible nature of cathepsin gene expression under normal conditions or during chemotherapy. We stimulated Human Embryonic Kidney (HEK293) cells and the human RCCC cell line 769P with increasing doses of Paclitaxel (Pac) and hydrogen peroxide (HP) and observed steady-state levels of *pro*-cathepsin S protein (CS, 37 kD) to be enhanced in a dose-dependent manner (Figure 1A), while p21 BAX protein levels were observed to diminish (Figure 1A, Supplementary Figure S1A,B).

In order to ascertain whether the changes in levels of CS (or even p21 BAX) proteins were due to transcriptional modulation of their genes, we performed quantitative RT-PCR analysis on total RNA isolated from cells stimulated in a dose-dependent manner using Pac or HP. CS and BAX transcripts were basally- or inducibly-expressed in HEK293 cells upon treatment with Pac (1–20 μg/mL) and HP (1–5 μM) (Figure 1B, Supplementary Figure S1C). In 769P cells, cathepsin S gene (*CTSS*) expression was induced with 1 μg/mL PAC and 0.1–5 μM HP (Figure 1B, Supplementary Figure S1D). *BAX* gene expression was also induced in 769P cells through stimulation with Pac at 1 μg/mL or HP at 0.1–1 μM (Figure 1B, Supplementary Figure S1D).

To confirm some of these effects, we embarked on analyzing the promoter region of the *CTSS* gene to ascertain whether it is amenable to inducible regulation under Pac or HP stimulatory conditions (Figure 1C). Using a GFP-protein reporter assay, the upstream -1048 bp fragment of the *CTSS* gene was inducibly responsive to stimulation of HEK293 or 769P cells with Pac or HP, as a respective 2–3 fold increase in GFP-reporter gene expression was observed (Figure 1D). Moreover, enhanced GFP-reporter expression could be clearly visualized under Pac stimulatory conditions in HEK293 cells (Figure 1E).

### 3.2. Expressed Cathepsin S Protein Destabilizes p21 BAX Protein Levels in Mammalian Cells

**Figure 2:**
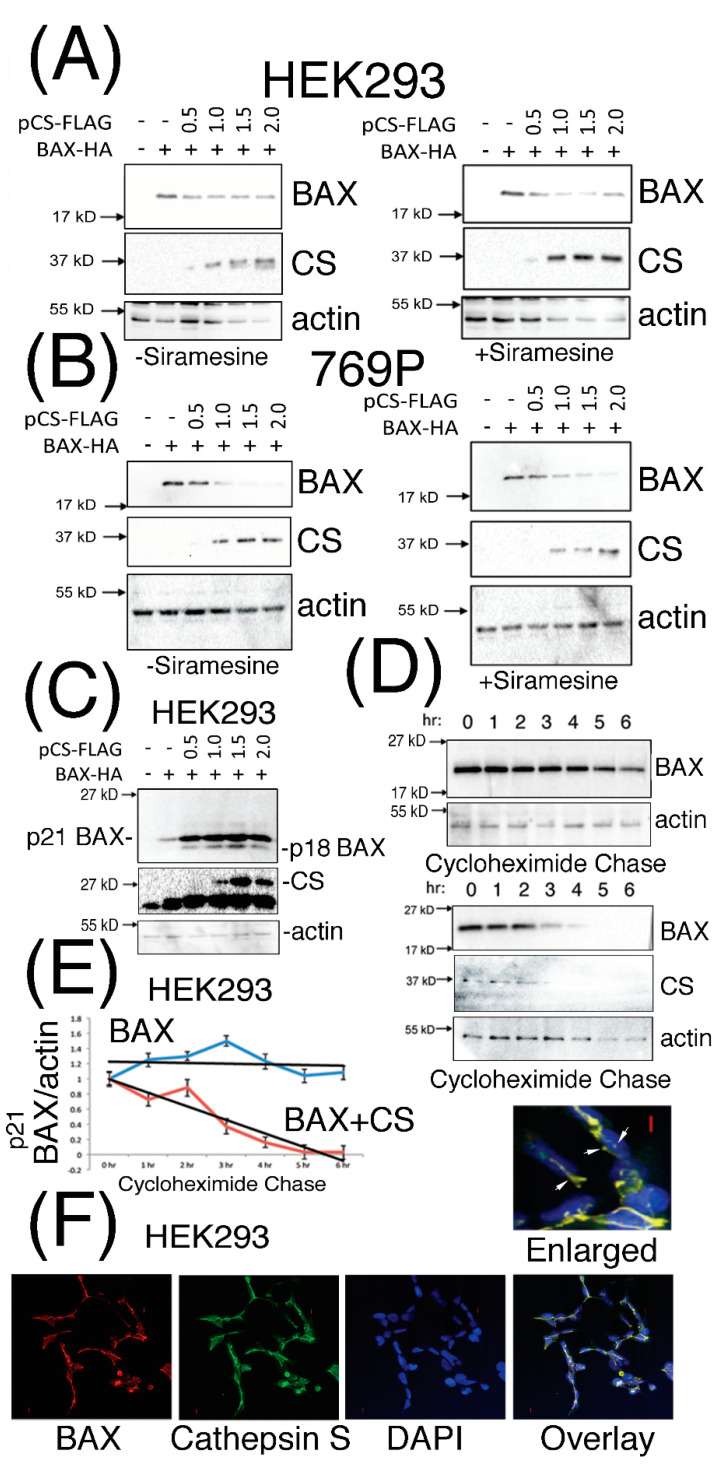
CS expression can destabilize the p21 BAX protein and co-localize with it in distinct cytoplasmic and peri-nuclear compartments. (Panels **A** and **B**), HEK293 and 769P cells were co-transfected with pBAX-HA and increasing doses of pCS-FLAG (μg) expression plasmids for 24 h, stimulated with carrier (left panels) or 2 μM Siramesine (right panels) for 1 h and equal volumes of soluble cell extracts analyzed for p21 BAX, CS and actin expression levels. (Panel **C**), HEK293 cells transfected for 48 h with the indicated μg amounts of plasmid DNA, were lysed and equal volumes of soluble extracts analyzed for BAX protein expression. (Panel **D**), Protein levels of p21 BAX were analyzed following cycloheximide pulse chase after HEK293 cells were transfected for 24 h with pBAX-HA (0.25 μg) alone or with pCS-FLAG (0.75 μg). p21 BAX expression levels were quantified, standardized for actin expression levels from cells treated with cycloheximide at T = 0 h. (Panel **E**), The blue and red curves indicate p21 BAX or p21 BAX levels upon co-expression with CS (respectively) and trend lines are shown in black. (Panel **F**), HEK293 cells co-expressing BAX-HA and CS for 24 h were stained for BAX and CS expression using *anti*-HA and -CS antibodies (SCB). BAX and CS co-localization is highlighted by white arrows and the red bars represent 1 micron. Quantified data is presented as the mean ± SEM and its significance (where *p* < 0.05) determined.

Previously published findings have proposed that destabilization of the p21 BAX protein can occur in a cathepsin protease-dependent manner in A-549, U-937, K-562 and HL-60 cells, and for which a cathepsin protease had not been identified and characterized [27]. Taken with our in silico analyses [34], we investigated the effect of co-expressing p21 BAX protein with increasing doses of CS protein expression in mammalian cells. Diminished steady state levels of p21 BAX protein were observed upon increasing levels of CS expression in HEK293 (Figure 2A) and 769P cells (Figure 2B), which were slightly reduced further upon the treatment of cells with the lysosomorphic agent Siramesine (Supplementary Figure S2A,B). Interestingly, under identical transfection conditions, an estimated p21 BAX-derived protein derivative of 18 kD was clearly visible following the incubation of transfected HEK293 for 48 h (Figure 2C). Moreover, CS-mediated destabilization of p21 BAX could be confirmed by performing cycloheximide pulse chase experiments in the presence or absence of CS with p21 BAX co-expression in HEK293 cells (Figure 2D,E). Moreover, CS and BAX proteins were observed to co-localize within distinct unidentified cytoplasmic and peri-nuclear filamentous structures (Figure 2F).

### 3.3. Cathepsin S-Mediated Cleavage of p21 BAX is Inhibited by the Novel CS Antagonist CS-PEP1 In Vitro and in Mammalian Cells

The approach for developing ‘BH3-mimetics’ through disrupting the interaction of the BH3 domain (from the *pro*-apoptotic BH3-only proteins) with the hydrophobic pocket of *anti*-apoptotic proteins does have demonstrated potential in effective and selective therapeutic design [35,36]. Being mindful of this approach, we identified a novel peptide (of sequence FFSFGGAL, called CS-PEP1) derived from the α5-helix from within the hydrophobic pocket of the Bcl-xl protein, based on the presence of this sequence in the fungal papain-like protease inhibitor, Pit2 [37]. We reasoned that such a peptide may exhibit three potentially useful qualities in that it may be inhibitory towards CS, while offering some degree of hindrance against inhibitory Bcl-xl interacting with p21 BAX (or other BH3-only proteins), in addition to it simultaneously blocking Bcl-xl homodimer formation through blocking the BH3-hydrophobic pocket interaction.

**Figure 3:**
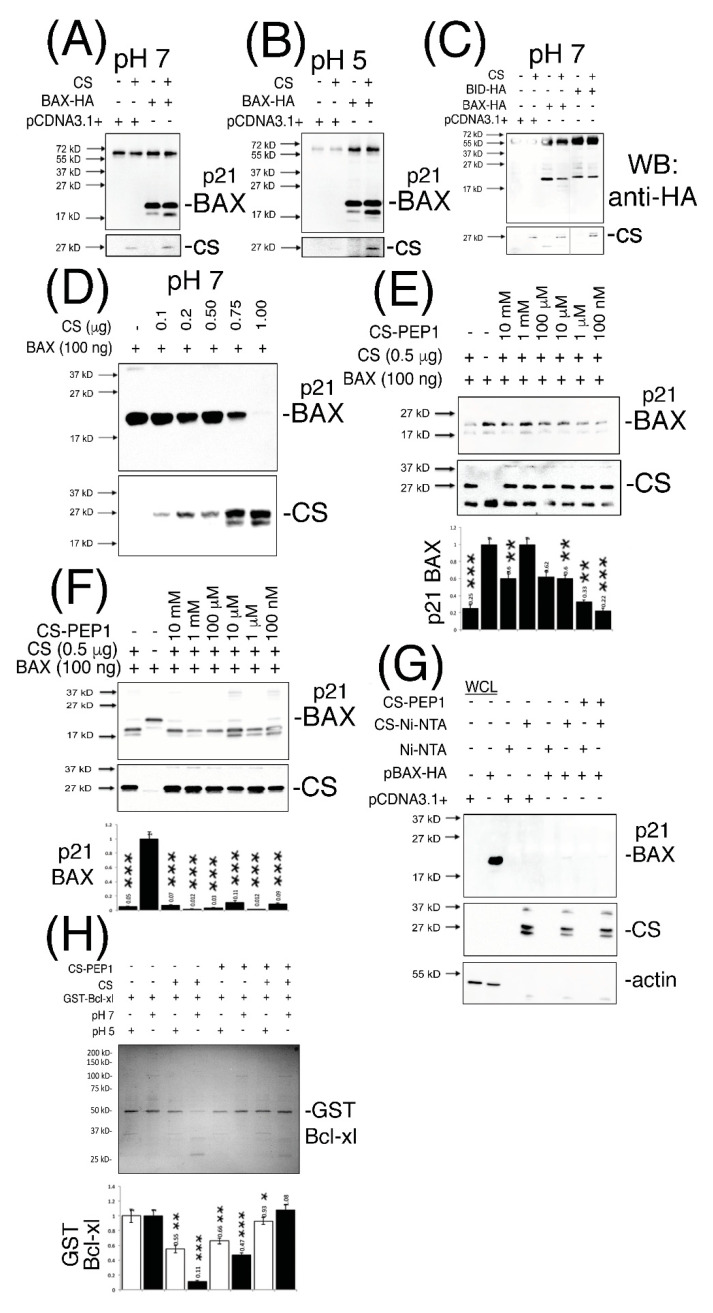
Active CS cleaves p21 BAX and GST-Bcl-xl in vitro and is inhibited by peptide CS-PEP1 in a pH-dependent manner. (Panels **A**–**C**), Equal cleared cell lysate volumes from HEK293 cells expressing BAX-HA or BID-HA for 24 h were immunoprecipitated using an *anti*-HA antibody, and washed immunoprecipitates exposed to active CS at pHs 7 and 5. Cleavage products were detected by Western blotting (top panels) and filters were re-probed to detect CS (lower panels). (Panel **D**), Equal quantities of bacterially expressed p21 BAX protein were exposed to active CS at pH 7 and the reactions analyzed for BAX cleavage using Western blotting using an *anti*-BAX antibody (Abcam). Filters were re-probed to detect CS (lower panels, SCB). (Panel **E**) BAX cleavage reactions at pH 7 were co-incubated in the presence of decreasing doses of CS-PEP1 and equal reaction volumes analyzed for p21 BAX cleavage products (top panel) and CS levels (middle panel), using an *anti*-BAX (Abcam) or *anti*-CS (SCB) antibody. p21 BAX substrate levels were quantified, standardized against BAX alone (lane 2) and are displayed in the lower panel. (Panel **F**), Equal amounts of immunoprecipitated p21 BAX-HA protein, derived from 0.25 μg BAX-HA expression in HEK293 cells, were co-exposed to active CS and decreasing amounts of CS-PEP1 at pH 7, and p21 BAX cleavage products (or CS) detected by Western blotting with an *anti*-BAX or -CS antibody (top and middle panels). p21 BAX substrate levels were quantified, standardized against BAX alone (lane 2) and are displayed in the lower panel. (Panel **G**), Cells expressing p21 BAX-HA were stimulated with carrier or CS-PEP1 (10 μM) for 24 h, lysed and equal volumes of soluble lysates (WCLs) incubated with activated CS-Ni-NTA agarose beads overnight (in the presence of 10 μM CS-PEP1), washed and analyzed for BAX-HA binding using an *anti*-HA antibody (top panel). CS (middle panel) and actin (lower panel) proteins were also revealed. (Panel **H**), In vitro CS-mediated cleavage assays were performed at pH 7 and 5 (top panel) using equal quantities of GST-Bcl-xl as a substrate in the presence of 100 μM CS-PEP1 (or carrier). Cleavage products were detected using Coomassie blue staining and substrate levels were quantified as shown in the lower panel (pH 7-white bars and pH 5-black bars). Quantified data are presented as the mean ± SEM and its significance (where *p* < 0.05) determined, using a two-way Student’s *t*-test (* *p* < 0.05, ** *p* < 0.01 and *** *p* < 0.001).

In striving towards addressing some of these key points, we firstly developed a CS-mediated ‘in vitro cleavage assay’ which confirmed that our bacterially expressed CS protein activated in vitro (and of size 27 kD) was catalytically-active against immunoprecipitated p21 BAX (Figure 3A,B) and BID protein substrates (Figure 3C) at pHs 7 and 5. Interestingly, enhanced p21 BAX-derived cleavage products of size 18- and 17-kD were readily discernable from cleavage reactions performed at pHs 7 and 5 (Figure 3A–C). As a novel substrate for CS, recombinant p21 BAX could also be cleaved in a CS dose-dependent manner at pH 7, as seen from (Figure 3D), and which could be inhibited by CS-PEP1 in the range of 10 mM–1 μM in vitro (Figure 3E, lanes 3–7). Similarly, CS-mediated cleavage of immunoprecipitated p21 BAX protein was significantly inhibited; through detection of the p21 BAX substrate or the accumulation of its 17- and 18-kD cleavage products (Figure 3F, lanes 3, 6, 8). Furthermore, CS-PEP1 dependent inhibition of CS-mediated p21 BAX cleavage did not appear to occur through the disruption of the CS-BAX protein interaction, as p21 BAX-HA could still interact with CS-Ni-NTA agarose beads in the presence of CS-PEP1 co-incubation (Figure 3G, lanes 6 and 8). As an important regulatory step for BAX protein activation, we also observed CS-PEP1 to exhibit inhibitory activity towards CS-mediated Bcl-xl cleavage in vitro (Figure 3H) at pH 7 (lane 8) significantly better than at pH 5 (lane 7). Of note, was the marginal destabilizing effects on GST-Bcl-xl protein at pH 5, and which may occur due to reductions in thermodynamic stability at this pH, as previously reported for the Bcl-xlΔTM protein derivative at pH 4.9, in vitro [38].

### 3.4. Dominant-Inhibitory Cathepsin S Expression and CS-PEP1-Mediated Cathepsin S Inhibition Rescues p21 BAX Destabilization

**Figure 4:**
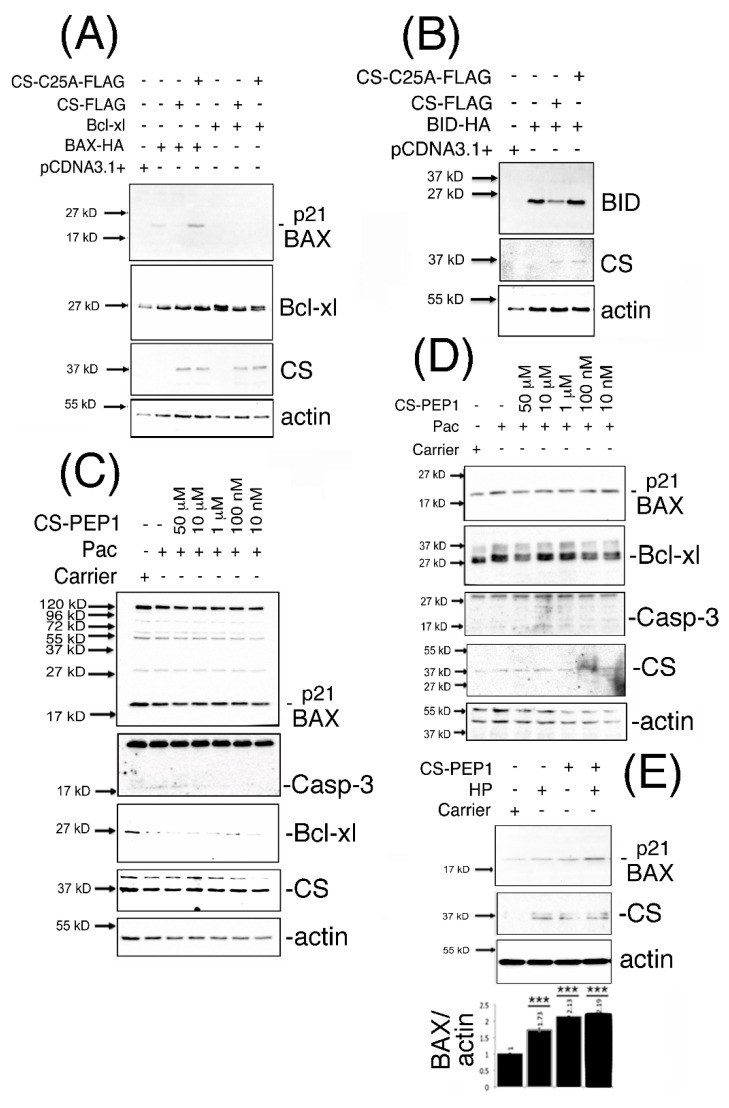
p21 BAX protein is stabilized in the presence of CS-C25A expression and in the presence of cell stimulation of HEK293 or 769P cells with CS-PEP1. HEK293 cells co-expressing BID-HA, Bcl-xl or BAX-HA with CS-FLAG or CS-C25A-FLAG for 24 h were lysed and equal volumes of soluble cellular extracts analyzed for p21 BAX, Bcl-xl or BID expression using Western blotting with *anti*-HA or *anti*-Bcl-xl antibodies (Panels **A** and **B**). HEK293 cells (Panel **C**) or 769P cells (Panel **D**) were co-stimulated with decreasing doses of CS-PEP1 in the presence of carrier or 5 μg/mL Pac for 24 h and equal volumes of cleared lysates analyzed for p21 BAX (Abcam), active Caspase-3 (Abcam), Bcl-xl (Abcam), CS (Invitrogen) and actin (Abcam) expression. 769P cells (Panel **E**) were co-stimulated with carrier or 5 μg/mL Pac, 10 μM zVAD-fmk and 10 μM CS-PEP1 for 24 h. Equal volumes of soluble whole cell lysates (WCLs) were analyzed for endogenous p21 BAX (Abcam), CS (Abcam) and actin (Abcam) expression. p21 BAX protein levels were quantified, standardized for actin expression and are represented by black bars as fold changes over stimulations with carrier alone. Quantified data are presented as the mean ± SEM and its significance (where *p* < 0.05) determined, using a two-way Student’s *t*-test (*** *p* < 0.001).

We next sought to address whether CS-mediated effects on BAX destabilization in mammalian cells could be mechanistically reversed, using the approaches of expressing dominant-inhibitory CS-C25A or stimulation of cells with CS-PEP1. Steady state levels of monomeric expressed p21 BAX, Bcl-xl and BID proteins (Figure 4A,B) were enhanced in the presence of expressed CS-C25A (Supplementary Figure S3A). Upon co-stimulation of HEK293 (Figure 4C) or 769P cells (Figure 4D) with CS-PEP1 and Pac, endogenous p21 BAX protein levels were enhanced between CS-PEP1 concentrations of 10 μM–10 nM (Supplementary Figure S3B,C). Interestingly, under Pac stimulatory conditions in HEK293 cells, Bcl-xl protein levels (in relation to p21 BAX protein levels) were observed to diminish (Supplementary Figure S3B). As reported in previous studies, this may be due to dose- and time-dependent responsiveness of cells to Pac, as seen with 8305C cells [39]. Moreover, down-regulation of Bcl-xl, has been reported upon the treatment of prostate cells with Docetaxel [40,41] or under oxidative stress conditions, in a Keap1 adaptor protein-dependent manner [42], and cannot be excluded as contributory factors. However, enhanced active caspase-3 (and diminished β-actin levels) levels were also evident in the presence of CS-PEP1 concentrations as low as 10 nM when stimulating HEK293 and 769P cells (Supplementary Figure S3C). Such findings are suggestive of apoptosis activation, in the presence of CS-PEP1 treatment of cells, and which can have the effect of masking gene regulatory events of importance. Consequently, co-treatment of 769P cells with the caspase inhibitor z-VAD (with Pac and CS-PEP1) did offer a more reliable alternative for visualizing enhanced CS-PEP1 mediated endogenous p21 BAX stabilization effects (Figure 4E), in the presence of β-actin stabilization and due possibly to effective cell death inhibition.

### 3.5. Knockdown Expression Cathepsin S and CS-PEP1-Mediated Cathepsin S Inhibition Rescues p21 BAX Destabilization

**Figure 5:**
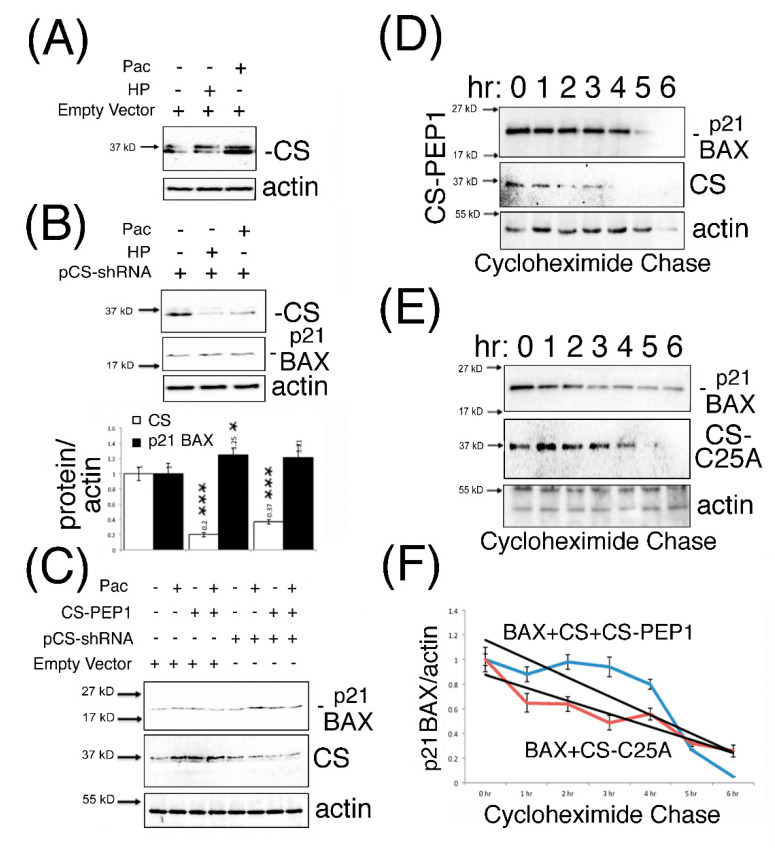
p21 BAX protein is stabilized in the presence of CS-shRNA expression, with or without CS-PEP1 co-treatment in HEK293 cells. HEK293 cells were transfected with 3 μg of empty vector (pTER, Panel **A**) or pCS-shRNA (Panel **B**) for 24 h, stimulated with Pac (5 μg/mL) or HP (1 μM) for a further 24 h and equal volumes of WCLs analyzed for endogenous CS (white bars) and p21 BAX (black bars) expression using Western blotting. Endogenous p21 BAX, CS and actin levels were detected from equal volumes of HEK293 soluble cell lysates by Western blotting, following 24 h of pCS-shRNA transfection (3 μg) and Pac (5 μg/mL) with CS-PEP1 (10 μM) co-stimulation (Panel **C**) for a further 24 hr. HEK293 cells co-transfected with pBAX-HA and pCS-FLAG or pBAX-HA and pCS-C25A-FLAG for 6 hr were stimulated with carrier or 10 μM CS-PEP1 for 18 h and treated with 20 μM cycloheximide. After 1 h, at the time points indicated, cells were lysed and equal volumes of soluble WCLs analyzed for p21 BAX, CS and actin expression using Western blotting (Panels **D**, **E**). Expression levels for p21 BAX were quantified, standardized for actin and expressed in relation to levels seen at T = 0 h. BAX and CS co-expression in the presence of CS-PEP1 stimulation (blue curve) and p21 BAX with CS-C25A co-expression (red curve) are indicated as trend lines (black curves, Panel 5**F**). Quantified data are presented as the mean ± SEM and its significance (where *p* < 0.05) determined, using a two-way Student’s *t*-test (* *p* < 0.05 and *** *p* < 0.001).

Previous findings have reported that CS expression is amenable to interference using shRNA approaches, as seen for CS-mediated regulation of the BRCA1 protein in breast cancer cells [30]. Consequently, we adopted a similar approach to ask whether the knockdown expression of CS could modulate steady-state endogenous p21 BAX protein levels, in the presence and absence of CS-PEP1 mediated inhibition of CS protease activity. As seen from Figure 5A, Pac- or HP-treatment of HEK293 cells could induce endogenous CS protein levels in HEK293 cells. Such an effect could also be abrogated in the presence of CS-shRNA expression, and from which enhanced p21 BAX protein levels were observed (Figure 5B). Furthermore, CS-PEP1 treatment appeared to show minimal synergistic effects on CS-knockdown-mediated steady-state p21 BAX protein levels in Pac treated HEK293 cells (Figure 5C). Lastly, the positive stabilizing effects of CS-PEP1 or CS-C25A on p21 BAX levels were supported further by cycloheximide pulse chase analyses, where p21 BAX when co-expressed with CS and CS-PEP1 treatment (Figure 5D) were seen to have similar protein stability levels to p21 BAX when co-expressed with CS-C25A (Figure 5E) over a 6 hr time course (Figure 5F).

### 3.6. Polyubiquitinated BAX (p120BAX) Is a Novel Substrate Targeted for Cleavage by Cathepsin S

**Figure 6:**
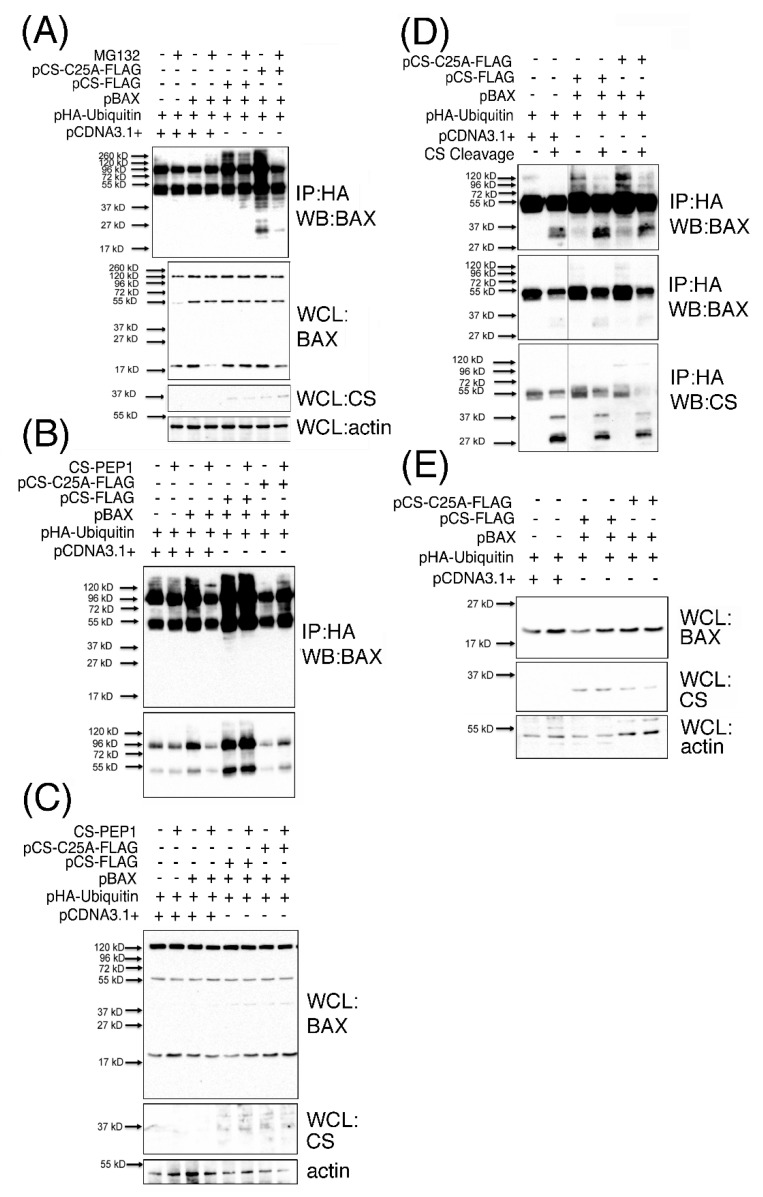
Ubiquitinated BAX protein species accumulate in the presence of pCS-C25A expression or CS-PEP1 stimulation of cells, and are cleaved by active CS in vitro. HEK293 cells expressing p21 BAX with CS-FLAG (or CS-C25A-FLAG) and HA-Ubiquitin for 24 h were treated with 10 μM MG132 or carrier for 5 h. Equal volumes of cleared whole cell lysates (WCLs) were immunoprecipitated (IP) using *anti*-HA antibodies and the immune complexes analyzed for BAX content using an *anti*-BAX antibody (Abcam) by Western blotting (WB). WCLs were also analyzed for BAX, CS (Invitrogen) and actin (Panel **A**). HEK293 cells co-expressing CS-FLAG or pCS-C25A-FLAG with BAX and HA-ubiquitin for 6 hr were treated further for 24 h with 10 μM CS-PEP1 and equal volumes of cleared WCLs immunoprecipitated using *anti*-HA antibodies. Immune complexes were analyzed for BAX protein using Western blotting at long- (Panel **B**, top panel) and short- exposures (Panel **B**, lower panel). Equal volumes of WCLs were also subjected to Western blotting to detect BAX, CS and actin expression levels (Panel **C**). HEK293 cells co-expressing BAX with CS-FLAG or CS-C25A-FLAG and HA-ubiquitin were lysed and equal volumes of cleared WCLs immunoprecipitated with *anti*-HA antibody. Immune complexes were washed and incubated in the presence of active CS at pH 7 and subjected to Western blotting to detect BAX-Ubiquitin-HA protein species (Panel **D**) after long- (top panel) or short-exposure times (middle panel). Here, CS expression was also detected by Western blotting (Panel **D**, lower panel). Equal volumes of WCLs were also subjected to Western blotting to detect BAX (Abcam), CS (Invitrogen) and actin (Abcam) expression (Panel **E**).

The accumulation of a number of BAX-derived protein species ranging in sizes 37–120-kD, which were readily detected from CS-PEP1 treated cells also captured our attention from Figure 4C, and which did not appear to correspond with the reported 80-, 96-, 160- or 260- kD homo-oligomerized sizes for the BAX protein [22]. Consequently, we hypothesized that the 120 kD BAX-derivative protein that was clearly visible from this data (Figure 4C) may represent a novel polyubiquitinated form of BAX arising in a CS-expression dependent manner. This was confirmed through identifying such a sized derivative amongst additional mono- and poly-ubiquitinated BAX derivatives, which were visible as a characteristic protein ‘ladder’ (or smear) in immunoprecipitates derived from cell extracts co-expressing p21 BAX, with CS or CS-C25A (Figure 6A), in the protein range from 27–260 kD. Briefly, polyubiquitinated BAX derivatives were detected upon BAX and CS co-expression (Figure 6A, lane 5) in comparison to BAX expression alone (Figure 6A, lane 3), implying that BAX polyubiquitination is CS expression-dependent. Under these expression conditions, and in the presence of MG132-mediated proteasomal inhibition, polyubiquitinated BAX derivative levels (Figure 6A, lane 4) were also enhanced in the presence of CS co-expression (Figure 6A, lane 6). Such findings suggest that BAX ubiquitination is CS expression-dependent and that the arising ubiquitinated BAX derivatives can also accumulate in the presence of proteasomal inhibition. Moreover, polyubiquitinated BAX derivatives were seen to accumulate upon *BAX* co-expression with CS-C25A (Figure 5A, lane 7) and less so in the presence of proteasomal inhibition (Figure 6A, lane 8), which would seem to suggest that the disposal of CS catalytic activity also permits the accumulation of polyubiquitinated BAX protein derivatives. For simplicity, we focused on the effects of CS-mediated accumulation of BAX ubiquitinated derivatives in the absence of proteasomal inhibition and observed the accumulation of 45–150 kD polyubiquitinated BAX species, upon basal CS inhibition in the presence of CS-PEP1 stimulation (Figure 6B, lane 4), or upon CS-C25A co-expression (Figure 6B, lane 8). Here, ubiquitinated BAX derivatives were readily observed in the presence of CS expression (Figure 6B, lane 5) and the levels of which could be marginally enhanced upon CS-PEP1 co-treatment of cells (Figure 6B, lane 6). These observations confirmed our previous findings (from Figure 6A) that positive CS protein expression (as seen from Figure 6C) permits the accumulation of ubiquitinated BAX derivatives and that the inhibition of CS activity also mediates the accumulation of such protein species, as seen with the presence of 120 kD polyubiquitinated BAX derivative (p120BAX). Based on these collective findings, we next asked whether the p120BAX polyubiquitinated derivative might be a substrate for active CS, *in vitro* (Figure 6D). Here, p120BAX was present in greater abundance upon CS-C25A and p21 BAX co-expression (Figure 6D, lane 5), in comparison to CS and p21 BAX co-expression (Figure 6D, lane 3) and that p120BAX could be cleaved by activated CS into three distinct polypeptides of approximate sizes 37, 38 and 45 kD, *in vitro* (Figure 6D). Moreover, these findings were supported by the positive expression of BAX and CS proteins in whole cell lysates (Figure 6E).

Collectively, such findings suggest that CS co-expression with BAX mediates BAX polyubiquitination and that in the presence of CS inhibition (in intact cells), BAX ubiquitinated protein levels are enhanced, and that one of these derivatives (p120BAX) can be cleaved by CS. These observations point to a scenario where the formation of BAX-ubiquitinated derivatives may require CS-mediated cleavage of p21 BAX as a prerequisite for the formation of polyubiquitinated BAX protein species (such as p120BAX), and which can be further cleaved by CS as a possible mechanism for CS-mediated depletion of cellular BAX protein levels.

### 3.7. Peptide CS-PEP1 Induces Apoptosis of HEK293 and 769P Kidney Cells

**Figure 7:**
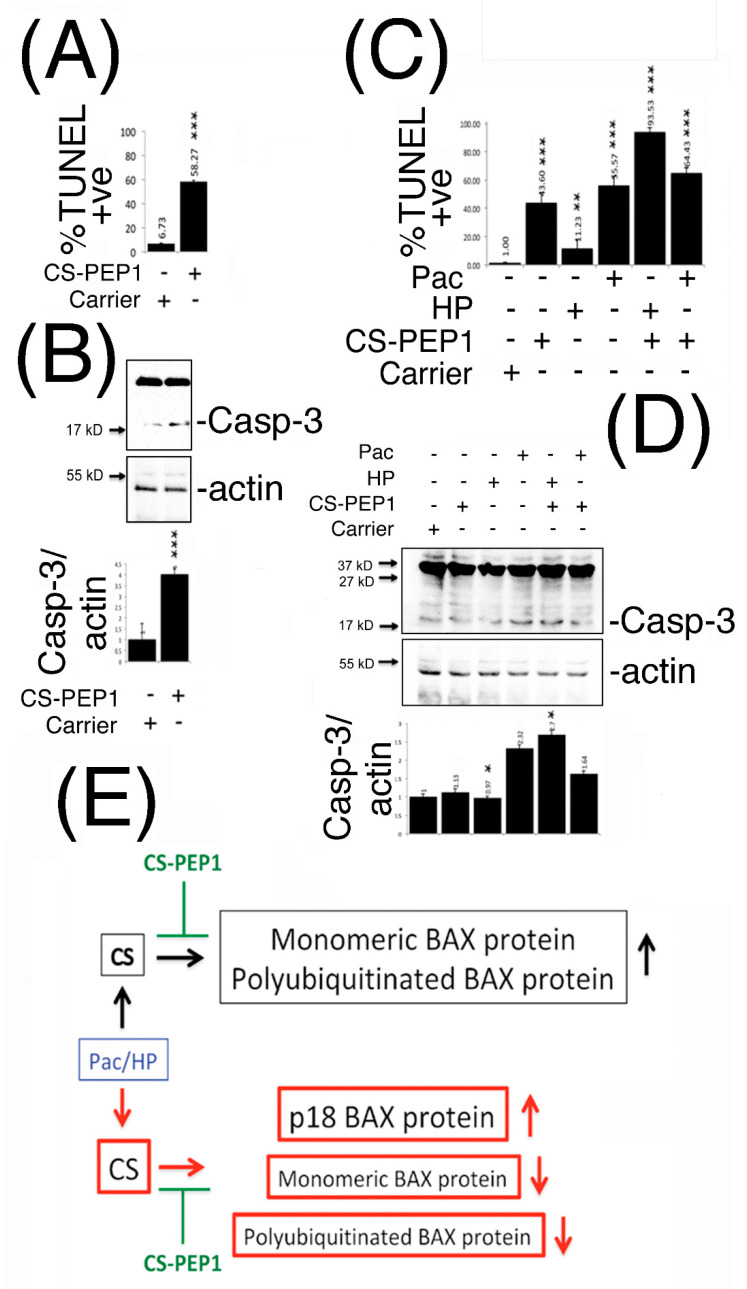
CS-PEP1 enhances apoptosis of HEK293 and 769P cells through enhanced caspase-3 activation, and interferes with a novel CS-mediated regulatory paradigm for the BAX protein. HEK293 (Panel **A**) or 769P cells (Panel **C**) were respectively co-stimulated with CS-PEP1 (10 μM), HP (5 μM) or Pac (5 μg/mL) for 24 h and apoptotic cells quantified after TUNEL staining. Equal volumes of soluble whole cell lysates (WCLs) prepared from cells treated identically as in Panels **A** and **C** were analyzed for active caspase-3 (Casp-3) and actin expression (Panels **B** and **D**). Expression levels of active caspase-3 were quantified and standardized for actin expression in relation to cell stimulated with carrier alone (Panels **B** and **D**, lower panels). (Panel **E**), At basal cathepsin S protein expression levels, BAX protein predominantly exists as a monomeric p21 form and which can undergo polyubiquitination-mediated destabilization (black boxes). Under Pac- and HP-stimulatory conditions (blue box), which induce *CTTS* gene expression, steady state levels of p21 monomeric BAX can be abrogated through the formation of a p18 kD BAX cleavage product and a p120kD-polyubiquitinated derivative of BAX (red boxes). Both axes of regulation can be negatively modulated by peptide CS-PEP1 (green), which consequently enhances cellular apoptosis, using a two-way Student’s *t*-test (* *p* < 0.05, ** *p* < 0.01 and *** *p* < 0.001).

In furthering our understanding of what input CS-PEP1 may have on modulating conventional chemotherapy treatment regimes, we next sought to determine the biological effects of CS-PEP1 (and CS-inhibition) on apoptosis of mammalian cells. Stimulation of HEK293 cells with CS-PEP1 increased apoptotic cell numbers approximately 10-fold, from 6 to 58% in a caspase-3 activation-dependent manner (Figure 7A,B and Supplementary Figure S4).

Similarly, treatment of 769P cells with CS-PEP1 alone enhanced apoptotic cell numbers from 1% to around 43%, while it also synergized the apoptotic-inducing effects of hydrogen peroxide from 11 to 93% (Figure 7C and Supplementary Figure S5). Paclitaxel-induced apoptosis was observed at around 55%, and for which CS-PEP1 treatment had the effect of increasing to 64% (Figure 7C), probably through the activation of caspase-3 (Figure 7D).

## 4. Discussion

Herein, we report that the *CTSS* (and *BAX*) genes are induced in response to Paclitaxel (Pac) or hydrogen peroxide (HP) treatments of kidney cells and that monomeric- and polyubiquitinated-BAX are novel substrates for the cysteine protease cathepsin S. Through the treatment of cells with the novel therapeutic peptide CS-PEP1, we could target cathepsin S protease activity, which gave rise to enhanced levels of monomeric p21 BAX protein in mammalian cells and in vitro. Additionally, CS-PEP1 showed promising activity in inducing apoptosis of HEK293 or 769P cells, either alone or in conjunction with Pac treatment. Collectively, such findings highlight a novel mechanism that connects *CTTS* expression with the intrinsic arm of the apoptotic pathway and which can be targeted for therapeutic purposes.

To support this model, the -1048 to -564 bp genomic DNA fragment upstream of the *CTSS* gene, showed positive transcriptional responsiveness to the effects of Pac and HP and the regulatory regions for which (until now) have remained unexplored [43,44,45,46,47,48]. Here, one transcription factor of relevance may be p53 as the predicted consensus binding sites for which [49] are located upstream of *CTSS* between -351 to -376 and -610 to -635 [50]. While p53 has also been suggested to contribute to lysosomal destabilization and apoptosis activation through its direct interaction with the lysosome [1,51,52], it may also contribute to lysosomal destabilization through it up regulating expression of the cathepsin D and cathepsin L genes [53,54,55].

As a central (and key) regulator of the intrinsic apoptosis pathway, whether p21 BAX is a substrate for cathepsin-mediated proteolysis has remained an area of controversy, with very little reported about which cathepsin member may be directly responsible for this [27]. When taken with additional findings which propose that total mitochondrial activated BAX protein levels are not equivalent to total cytosolic BAX protein (in apoptotic cells), the emphasis placed on p21 BAX cleavage as a possible key regulatory step during apoptosis has held high importance [22,56]. As reported herein, our findings are in good agreement with this latter line of research, as *CTSS* and *BAX* genes were inducibly expressed in kidney cells and from which concomitant increases in their transcriptional products were not reflected at the protein level. From our observations, CS could cleave p21 BAX in vitro and in mammalian cells, to yield cleavage intermediate products of sizes 18 and 17 kD, and which support earlier reports where the cleavage of p21 BAX at Asp-33 can give rise to increased p18 BAX levels, which enhance apoptosis and possess a very short half-life [27]. In this latter context, published findings that BAX can be localized in a perinuclear compartment [57], particularly under conditions that favor proteasomal inhibition [58] are in strong agreement with our observations as possibly being a unique subcellular compartment where BAX (and CS) can co-exist. From our findings, this has particular relevance as seen through the pH-dependency with which CS-mediated cleavage of p21 BAX (or its polyubiquitinated derivatives) can occur and which might have regulatory significance if this perinuclear compartment is lysosomally-derived. This could be important, as CS appears to have varying degrees of specificity towards our Bcl-xl substrate in a pH-dependent manner (Figure 3). Whereas CS-PEP1 inhibits CS-mediated p21 BAX and GST-Bcl-xl cleavage at pH 7, at the lower pH of 5 it inhibits CS-mediated p21 BAX cleavage better than CS-mediated Bc-xl cleavage, in vitro. This level of pH selectivity that CS-PEP1 inhibitory activity is associated with could therefore highlight a novel regulatory mechanism, and which may be exploited for better therapeutic design and substrate-specific inhibition.

Based on the above novel findings, targeting CS indeed takes on greater significance, as the inhibition of CS activity may permit p21 BAX stabilization so that it may fulfill its role as key activator of the intrinsic arm of the apoptotic pathway. While, CS-PEP1 appears to be inhibitory to cell growth at concentrations ranging from millimolar- to nanomolar- quantities, from our findings it appears to mechanistically have the effect of stabilizing p21 BAX protein levels in proportion to Bcl-xl protein levels in intact cells. Consequently, the rates at which cells undergo apoptosis based upon how sensitized they may have become upon exposure to CS-PEP1, does take on greater relevance and warrants further mechanistic clarification. Moreover, such factors do highlight important considerations for potentially permitting CS-PEP1 efficacy (or selectivity) to be modulated with greater effect in the context of characterizing it for further therapeutic development.

While the Parkin [59] and Ku70 [60] proteins are respective E3-ligase and DUB proteins for BAX, how CS interplays with their activity dynamics is a good question that has arisen from our findings. Whereas p47-, p55-species of ubiquitinated BAX have been reported in the past, their presence was positively correlated with a greater number of apoptotic cells [58] through the localization of ubiquitinated BAX to mitochondria. Whether these ubiquitinated BAX derivatives arise in a CS-dependent manner, either through partial ubiquitination and enhanced DUB activity, or a combination of these factors remains currently unknown. Nevertheless, our findings do support an emerging role for cathepsin S in the regulation of pathologically relevant proteins through the ubiquitination pathway, as an association that has been suggested previously in the instance of CS facilitating the ubiquitin dependent degradation of BRCA1 [30].

In summary, we propose that p21 BAX and polyubiquitinated BAX are novel substrates for the cysteine protease, CS. Additionally, CS activity can be targeted by CS-PEP1, which may enhance drug-mediated apoptosis of kidney cells through interfering with CS and the p21 BAX-Bcl-xl regulatory axis of the intrinsic pathway for apoptosis (Figure 7E).

## 5. Conclusions

In conclusion, Cathepsin S or BAX gene expression is induced upon the treatment of human kidney cell lines with Paclitaxel or Hydrogen Peroxide stimulation. p21 BAX and ubiquitinated BAX derivatives are novel substrates for activated cathepsin S *in vitro* or within intact mammalian cells, and is a reaction that can be therapeutically targeted by the novel therapeutic, CS-PEP1. Finally, CS-PEP1 can mechanistically induce apoptosis of kidney cells through BAX stabilization, Bcl-xl destabilization, cathepsin S inhibition, or a combination of these effects.

## 6. Patents

There is a patent resulting from this work.

## Data Availability

The data presented in this study are available on request from the corresponding author. The data is not publicly available due to patent protection being sought for some of this work.

## Supplementary Materials

The following are available online at https://www.mdpi.com/1999-4923/13/3/339/s1. Figure S1: Quantification of CS and p21 BAX expression in HEK293 or 769P cells under Pac or HP stimulatory conditions; Figure S2: Quantification of p21 BAX expression in HEK293 or 769P cells under increasing levels of CS co-expression; Figure S3: Quantification of p21 BAX protein in the presence of CS-C25A expression or upon stimulation of HEK293 and 769P cells; Figure S4: CS-PEP1 stimulation induces apoptosis of HEK293 cells; Figure S5: CS-PEP1 stimulation induces apoptosis of 769P~cells.
