# Supplementary material for: Cathepsin S Cleaves BAX as a Novel and Therapeutically Important Regulatory Mechanism for Apoptosis
Source: Pharmaceutics. 2021 Mar 5;13(3):339. doi: 10.3390/pharmaceutics13030339 (PMC8035670; doi:10.3390/pharmaceutics13030339)
Supplement: Supplementary file 1 [file pharmaceutics-13-00339-s001.pdf]

pharmaceutics

MDPI

Supplementary Materials: Cathepsin S Cleaves BAX as a Novel and Therapeutically Important Regulatory Mechanism for Apoptosis

Surinder M Soond*, Lyudmila V. Savvateeva, Vladimir A. Makarov, Neonila V. Gorokhovets, Paul A. Townsend and Andrey A. Zamyatnin Jr.*

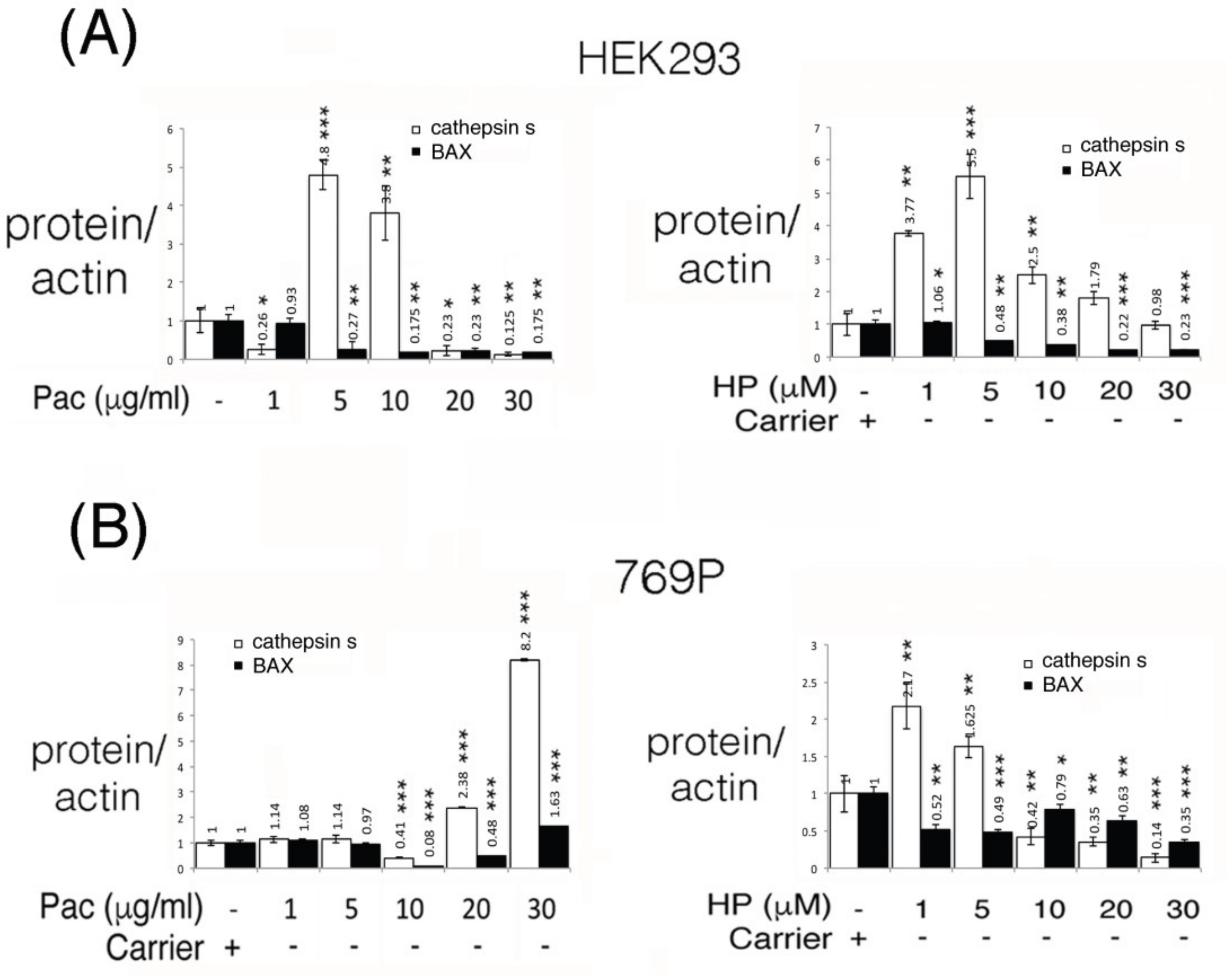

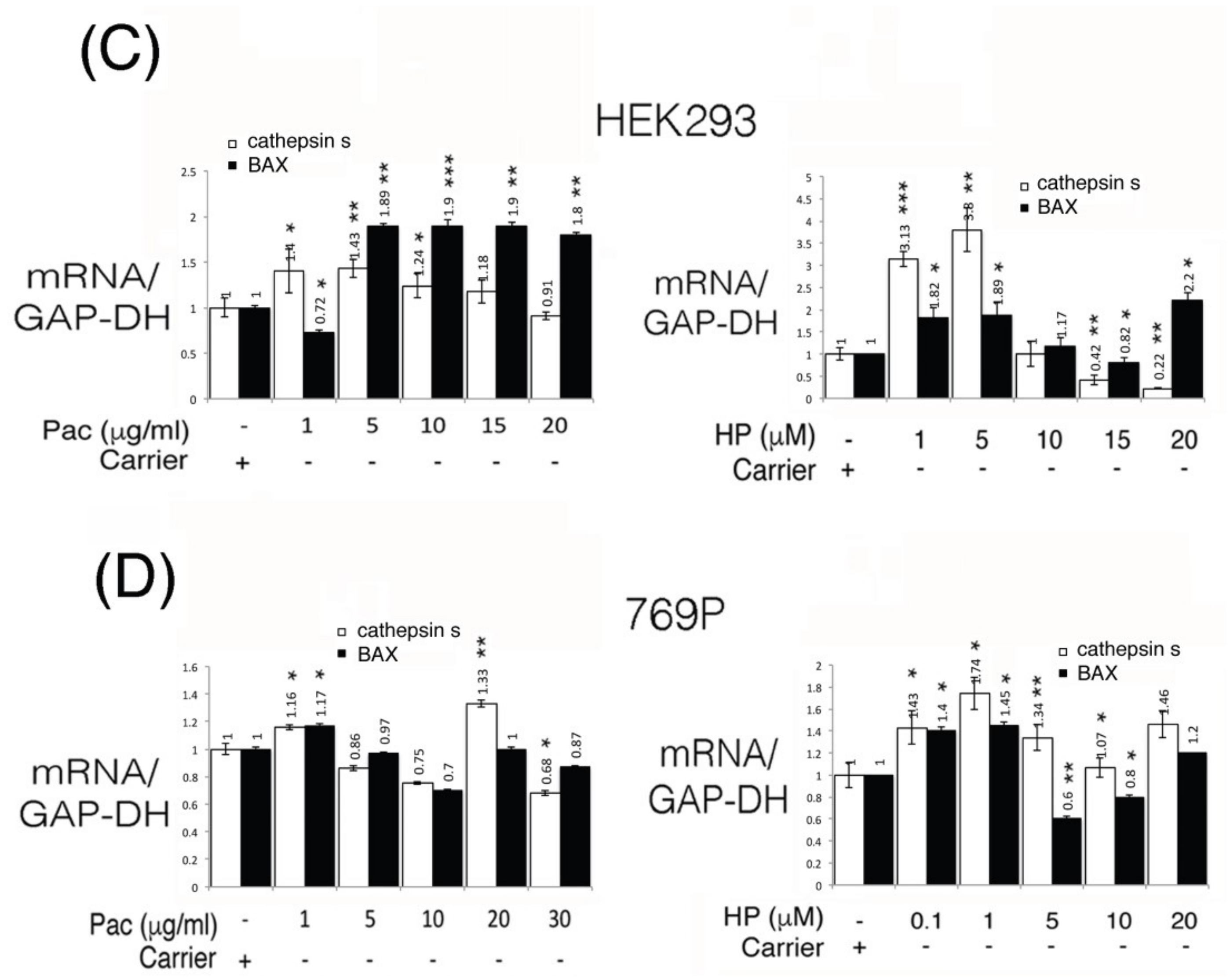

Figure S1. Quantification of CS and p21 BAX expression in HEK293 or 769P cells under Pac or HP stimulatory conditions. (Panels A and B), HEK293 and 769P cells were stimulated with increasing doses of Paclitaxel (Pac) and Hydrogen Peroxide (HP), equal volumes of soluble lysates prepared after 24 h and analyzed by Western blotting for cathepsin S (CS), p21 BAX and actin expression (from Figure 1). Expression levels were quantified and standardized for β-actin expression levels, and are displayed as fold changes over cells stimulated with carrier alone. The white and black bars represent CS and p21 BAX expression levels, respectively. (Panels C and D), Total RNA was isolated from cells stimulated with increasing doses of Pac or HP and equal quantities of template cDNA analyzed for CS, BAX and Glyceraldehyde-3-phosphate dehydrogenase (GAP-DH) transcript expression (from Figure 1), using semi-quantitative RT-PCR. Expression levels were quantified, standardized for GAP-DH expression, and displayed as fold expression level changes over cells stimulated with carrier alone. White and black bars represent CS and p21 BAX expression levels, respectively. Quantified data is presented as the mean ± SEM and its significance (where $p < 0.05$) determined, using a two-way Student t-test (*$p < 0.05$, **$p < 0.01$ and ***$p < 0.001$).

Publisher's Note: MDPI stays neutral with regard to jurisdictional claims in published maps and institutional affiliations.

Copyright: © 2021 by the authors. Submitted for possible open access publication under the terms and conditions of the Creative Commons Attribution (CC BY) license (http://creativecommons.org/licenses/by/4.0/).

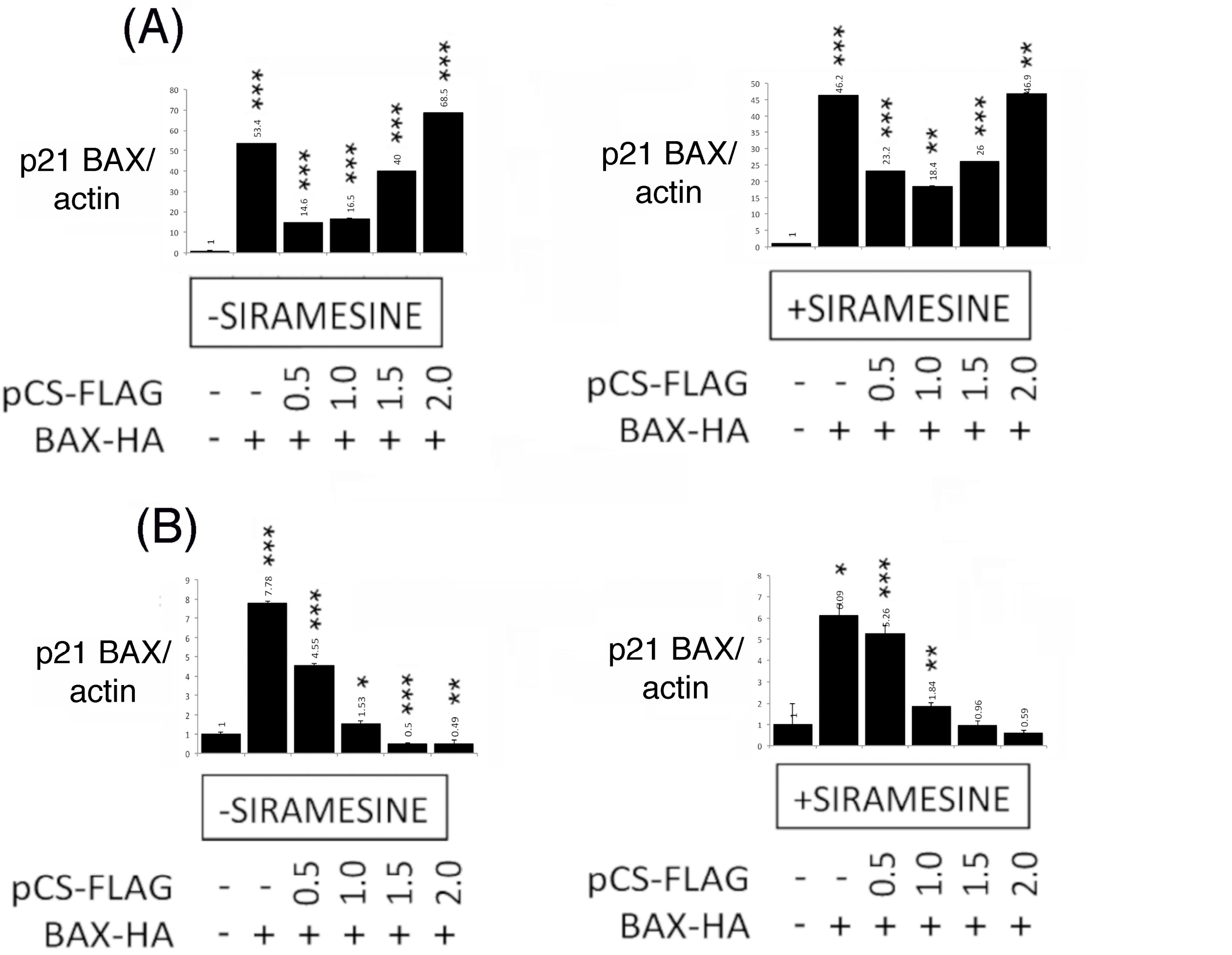

Figure S2. Quantification of p21 BAX expression in HEK293 or 769P cells under increasing levels of CS co-expression. HEK293 (Panel A) and 769P (Panel B) cells were co-transfected with pBAX-HA and increasing µg doses of pCS-FLAG expression plasmids for 24 hr, stimulated with carrier (left panels) or 2 µM Siramesine (right panels) for 1 hour and equal volumes of soluble cell extracts analyzed for p21 BAX, cathepsin S (CS) and β-actin expression levels using Western blotting (from Figure 2). BAX expression levels were quantified, standardized for β-actin expression and normalized against cells transfected with empty vector alone. Quantified data is presented as the mean ±SEM and its significance (where $p < 0.05$) determined, using a two-way Student t-test (*$p < 0.05$, **$p < 0.01$ and ***$p < 0.001$).

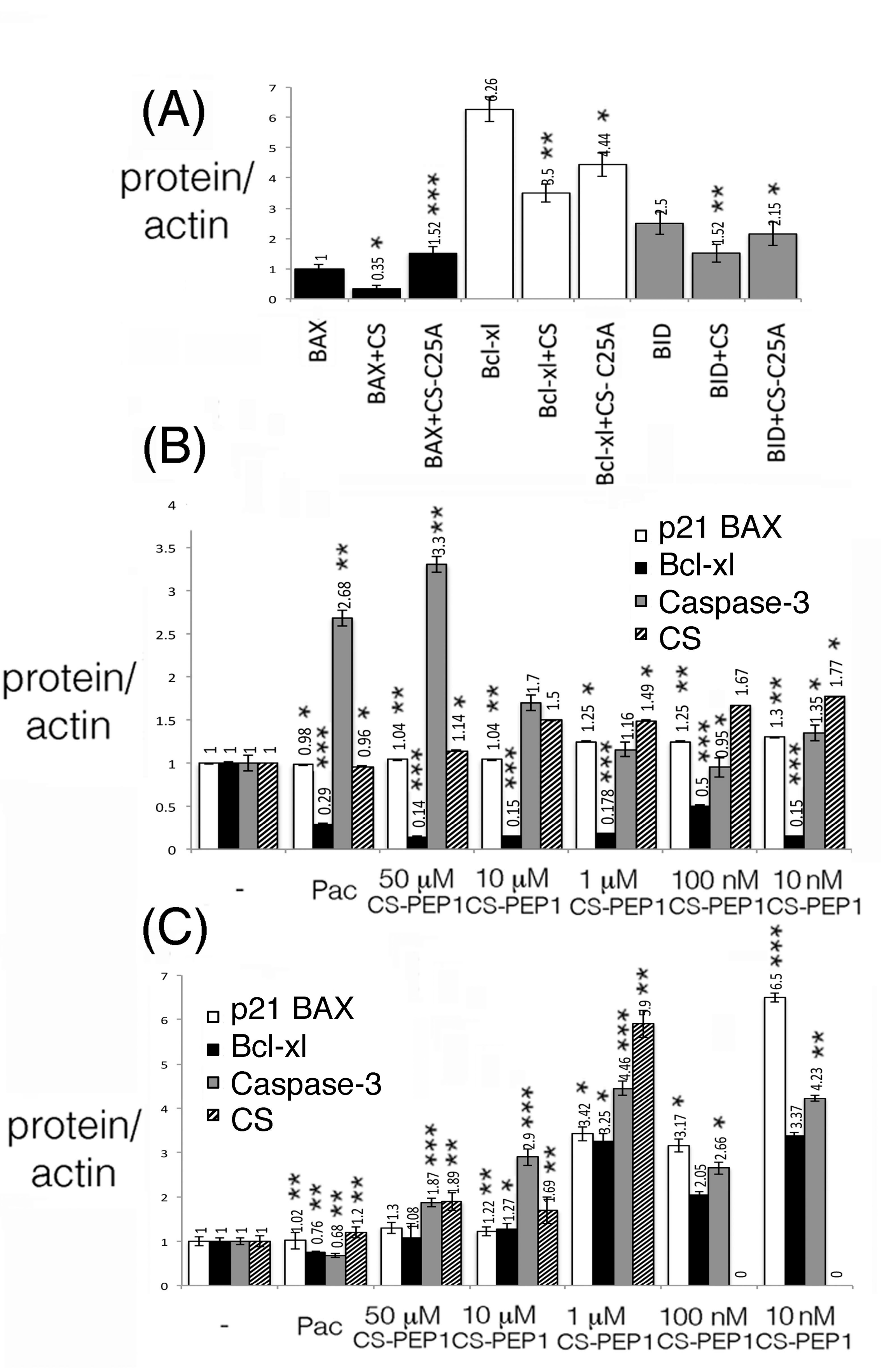

Figure S3. Quantification of p21 BAX protein in the presence of CS-C25A expression or upon stimulation of HEK293 and 769P cells. (Panel A) p21 BAX (black bars), Bcl-xl (white bars) or BID (grey

bars) protein levels were quantified following their 24 hr co-expression with CS-FLAG or CS-C25A-FLAG in HEK293 (from Figure 4 Panels A and B). Expression levels were standardized for β-actin expression and presented as fold expression over cells transfected with empty vector alone (pCDNA3.1+). HEK293 cells (Panels B) or 769P cells (Panels C) were stimulated with decreasing doses of CS-PEP1 in the presence of carrier or 5 μg/ml Pac for a further 24 hours (from Figure 4, Panels C and D) and quantified for endogenous p21 BAX (Abcam), Bcl-xl (Abcam), active caspase-3 (Abcam) and CS (Invitrogen) expression. Expression levels of these proteins were standardized for β-actin expression and are presented as fold expression over cells stimulated with carrier alone by white, black, grey and hatched bars. Quantified data is presented as the mean ±SEM and its significance (where $p < 0.05$) determined, using a two-way Student *t*-test (*$p < 0.05$, **$p < 0.01$ and ***$p < 0.001$).

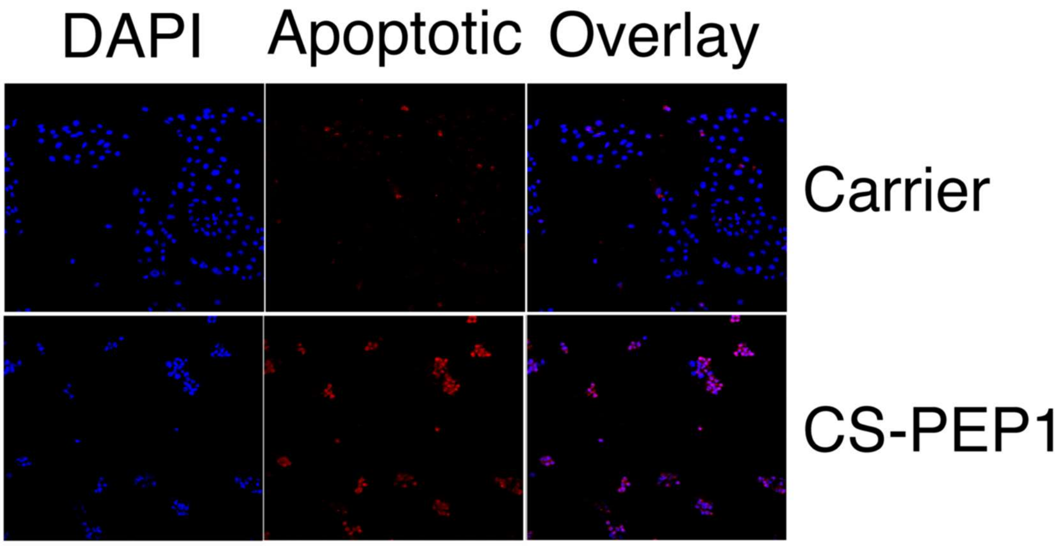

Figure S4. CS-PEP1 stimulation induces apoptosis of HEK293 cells. HEK293 cells were stimulated with 10 M CS-PEP1 or carrier for 24 h and apoptotic cells quantified using TUNEL staining (red) and nuclear DAPI counter staining (blue).

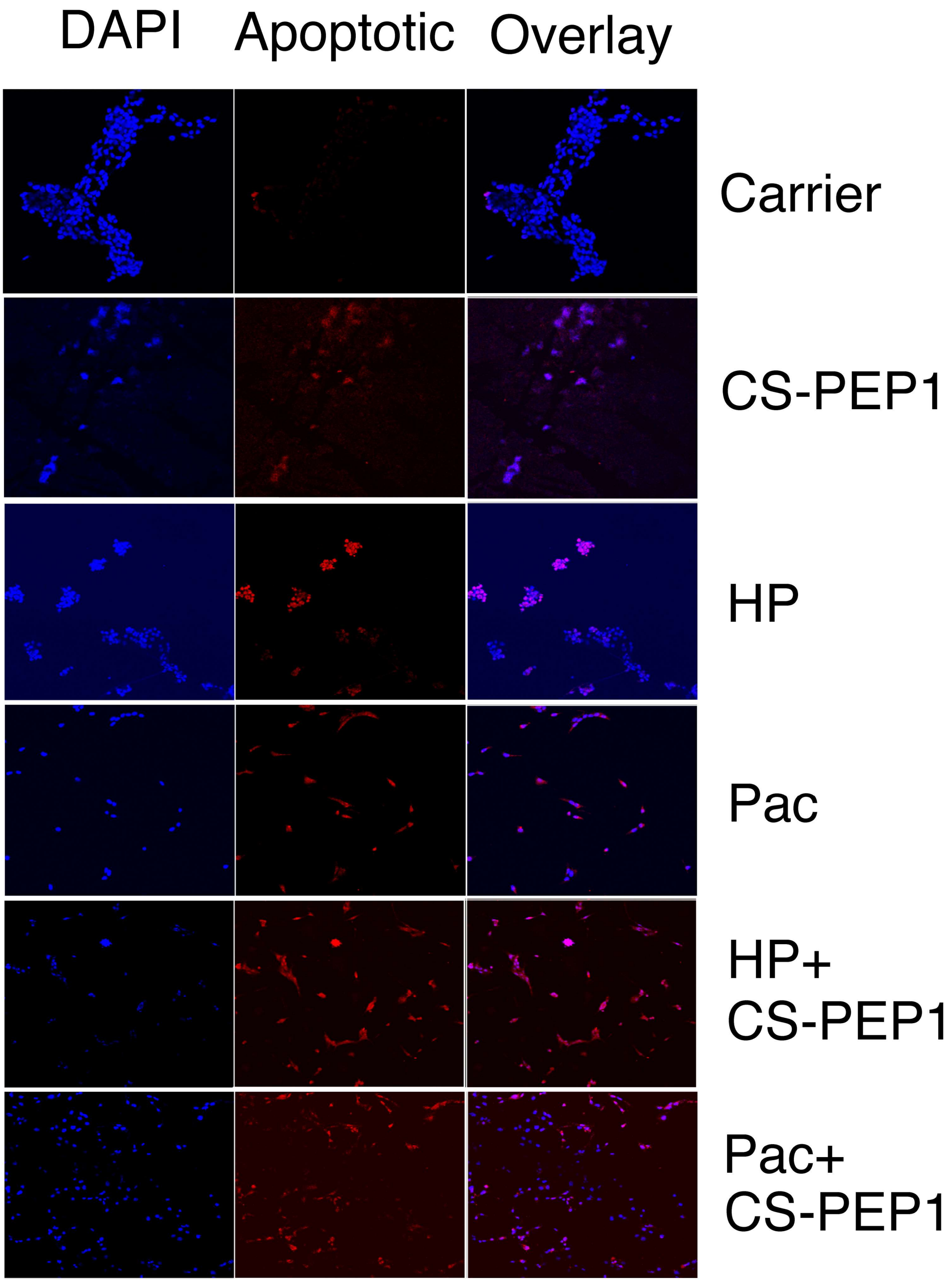

Figure S5. CS-PEP1 stimulation induces apoptosis of 769P cells. 769P cells were co-stimulated with CS-PEP1 (10 µM), and HP (5 µM) or Pac (5 µg/mL), for 24 h and apoptotic cells quantified using TUNEL staining (red) and nuclear DAPI counter staining (blue).
